# Metastatic Follicular Thyroid Carcinoma Secreting Thyroid Hormone and Radioiodine Avid without Stimulation: A Case Report and Literature Review

**DOI:** 10.1155/2014/584513

**Published:** 2014-10-07

**Authors:** Syed A. Abid, Brendan C. Stack, Donald L. Bodenner

**Affiliations:** ^1^Department of Geriatrics, University of Arkansas for Medical Sciences, 4301 W Markham Street, Little Rock, AR 72205, USA; ^2^Department of Otolaryngology-Head and Neck Surgery, Thyroid Center, University of Arkansas for Medical Sciences, 4301 W Markham Street, Little Rock, AR 72205, USA; ^3^Department of Geriatrics, Department of Otolaryngology-Head and Neck Surgery, Thyroid Center, University of Arkansas for Medical Sciences, 4301 W Markham Street, Little Rock, AR 72205, USA

## Abstract

*Introduction*. This is an extremely rare case of a patient with metastatic follicular thyroid cancer who continued to produce thyroid hormone and was iodine scan positive without stimulation after thyroidectomy and radioiodine (I-131) therapy. *Patient Findings*. A 76-year-old Caucasian male was diagnosed with metastatic follicular thyroid carcinoma on lung nodule biopsy. Total thyroidectomy was performed and he was ablated with 160 mCi of I-131 after recombinant human thyrotropin (rhTSH) stimulation. Whole body scan (WBS) after treatment showed uptake in bilateral lungs, right sacrum, and pelvis. The thyroglobulin decreased from 2,063 to 965 four months after treatment but rapidly increased to 2,506 eleven months after I-131. Thyroid stimulating hormone (TSH) remained suppressed and free T4 remained elevated after I-131 therapy without thyroid hormone supplementation. He was treated with an additional 209 mCi with WBS findings positive in lung and pelvis. Despite I-131, new metastatic lesions were noted in the left thyroid bed and large destructive lesion to the first cervical vertebrae four months after the second I-131 dose. *Conclusions*. This case is exceptional because of its rarity and also due to the dissociation between tumor differentiation and aggressiveness. The metastatic lesions continued to secrete thyroid hormone and remained radioiodine avid with rapid progression after I-131 therapy.

## 1. Introduction

Follicular thyroid cancer (FTC) is the second most common type of thyroid malignancy worldwide after papillary thyroid cancer [1]. Its incidence, however, is higher than papillary thyroid cancer in geographic areas of endemic goiter, accounting for 25%–40% of cases in areas of iodine deficiency compared to 10% of all cases of thyroid malignancy in iodine-sufficient areas [1]. In the United States, the incidence of follicular thyroid cancer is lower due to adequate dietary iodine [1].

Thyroid cancer incidence has doubled since 1990 and it is estimated that 60,000 new cases will be diagnosed this year though the mortality rates remain stable [2]. It is more prevalent in women and is usually diagnosed at an earlier age compared to other adult cancers [2]. Follicular and papillary types of thyroid cancers are classified as differentiated and have a better prognosis compared to the anaplastic form, which is undifferentiated and more aggressive [3]. Peak incidence of follicular thyroid cancer in the US is between 30 and 60 years of age, with a female to male ratio of 3 : 1 [2].

Thyroid cancer usually presents as an asymptomatic solitary thyroid nodule that may be palpable on physical exam but is more likely to be identified incidentally on CT or ultrasonography. The definitive diagnosis is a biopsy which helps to differentiate an adenoma from cancer [3]. It may, in rare cases, present as metastatic disease mainly to the lung and bone; however, it can also metastasize to other areas including the brain, liver, and skin [3]. The treatment usually involves thyroidectomy followed by radioactive iodine to ablate the remaining tissue and any metastatic sites [3]. Well-differentiated cancers are seldom aggressive and aggressive cancers rarely have the capacity to produce hormone. Metastatic follicular cancer producing thyroid hormone remains extremely rare with only 47 cases reported from 1946 to 2005 as noted by Tardy et al. [4].

This is an extremely rare case of a patient who underwent thyroidectomy and radioiodine therapy for an aggressive metastatic thyroid cancer, yet he remained euthyroid as the metastatic tumor cells retained the ability to produce thyroid hormone. A review of the literature on the possible explanation of hormone production by metastatic thyroid cancer and the factors involved in tumor metastasis follows the case presentation.

## 2. Case Report

A 76-year-old Caucasian male was referred to our clinic for follow-up after a diagnosis of follicular thyroid cancer. The following history and workup were extracted from the patients' medical records prior to his visit to our clinic. The thyroid cancer diagnosis was made incidentally after a chest computerized tomography (CT) scan, done in early 2009 for a workup of dyspnea, showed multiple noncalcified lung nodules bilaterally and an adrenal mass of 1.5 cm. A workup of metastatic cancer was conducted, which included a CT of the neck that showed a multinodular goiter but no lymphadenopathy. A positron emission tomography-computerized tomography (PET-CT) scan was also performed, which did not reveal any nodes or nodules with increased uptake. A CT guided biopsy of the lungs was nondiagnostic. In May 2009, a thyroid scan was done, which showed diffuse uptake in the thyroid (46% at 24 hours), and he was treated with I-131 at that time. In March 2010, a repeat CT of the chest showed an increase in number and size of the lung nodules. A subsequent lung biopsy showed follicular appearing cells with glandular structure. The histological diagnosis was made with positive staining of tissue for thyroglobulin and thyroid transcription factor 1 (TTF-1). Thyroid labs from June 25, 2010, showed suppressed TSH 0.01 mIU/L and free T4 of 1.57 ng/dL.

**Figure 1:**
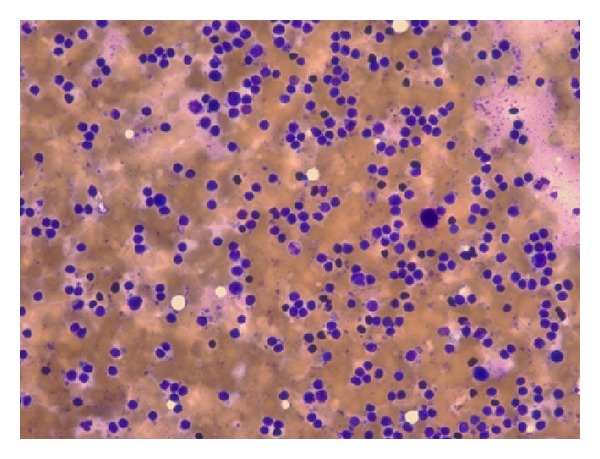
Fine needle aspirate (FNA) of lymph node from left neck. Diagnosis negative for malignancy. Favor reactive lymph node.

The patient was seen in our clinic for the first time on June 30, 2010. Physical exam findings were noncontributory and the patient did not have shortness of breath, tremors, palpitations, heat or cold intolerance, constipation, diarrhea, weight changes, or fatigue. He did not have a family history of thyroid disorders. On neck exam there was a 3 cm mass felt on the left when the patient was asked to swallow. An ultrasound of the thyroid revealed a small right lobe with some scarring and hyperechogenicity in the bed which may be a result of radioiodine therapy. On the left lobe there was a large poorly encapsulated hypoechoic complex cystic lesion with calcifications measuring approximately 4.0 cm × 3.0 cm × 3.8 cm. Along with this lesion there were some subcentimeter lymph nodes found on ultrasound, which also raised suspicion for malignancy. The assessment and plan included fine needle aspiration (FNA) biopsy of the lymph nodes and a total thyroidectomy with possible lateral neck dissection depending on the biopsy results. The lymph node FNA came back negative for malignancy (Figure 1); however, since the diagnosis of metastatic thyroid cancer had been reached from the CT guided lung biopsy, the patient was scheduled for a total thyroidectomy.

## 3. Postsurgery History

**Figure 2:**
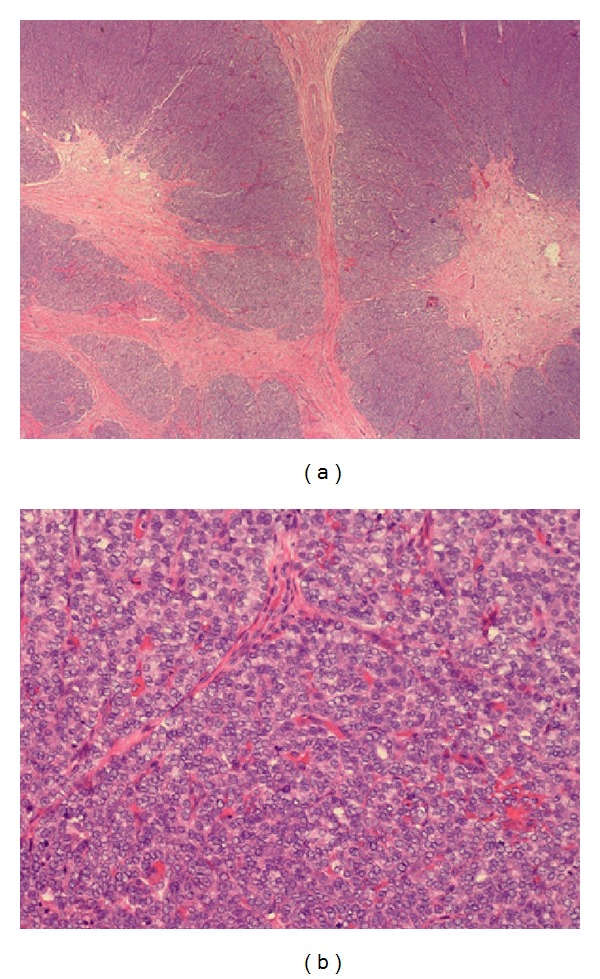
(a) Thyroid, total thyroidectomy. Widely invasive follicular carcinoma, 4.5 cm, with lymphovascular invasion and extracapsular extension. Negative margins. (b) Lymph node negative for malignancy.

**Figure 3:**
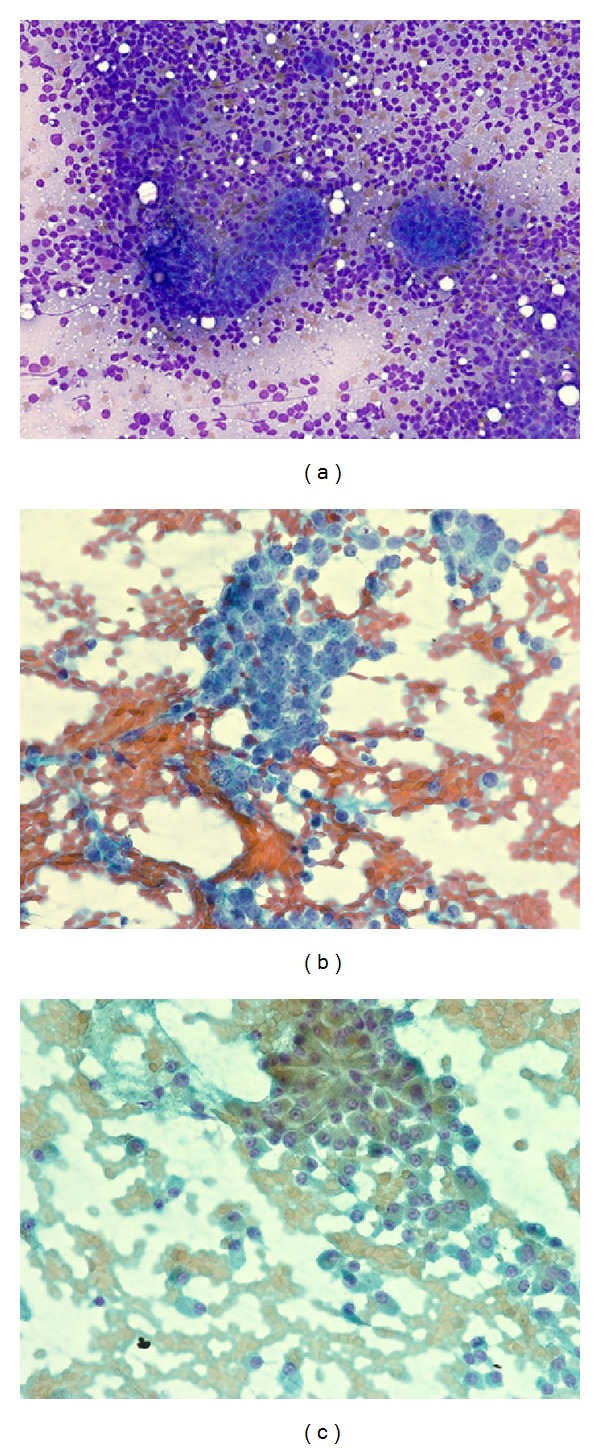
(a) Lymph node FNA showing metastatic follicular carcinoma. (b) Lymph node FNA showing metastatic follicular carcinoma. (c) Lymph node FNA showing metastatic follicular carcinoma.

The patient had a total thyroidectomy on July 30, 2010, two months after his cancer diagnosis. The postsurgery pathology report revealed a left lobe widely invasive follicular carcinoma (Figure 2(a)). The lesion measured 4.5 cm in greatest diameter with lymphovascular invasion and extracapsular extension. A lymph node dissection was negative for malignancy (Figure 2(b)). The right lobe had a benign multinodular goiter. Approximately a month after the surgery, on August 25, 2010, the patient underwent radioactive iodine (RAI) ablation with 160 mCi of I-131 after rhTSH stimulation. Post-RAI ablation, whole body scan, and single-photon emission computerized tomography (SPECT) scan were acquired on September 3, 2010. The scans showed focal uptake in the thyroid bed region as well as multiple foci of uptake throughout both lung fields and small lytic lesions in the right sacrum and left anterior iliac bone. Physiologic uptake was seen in the liver and bladder. Thyroid labs on September 29, 2010, showed that TSH was still suppressed at 0.01 mIU/mL and free T4 was slightly elevated at 1.86 ng/dL. Thyroglobulin decreased from 2,063 ng/mL to 965 ng/mL four months after treatment but rapidly increased to 2,506 ng/mL seven months later.  Figure 7 shows a brief summary of the changes in thyroglobulin and TSH values during course of treatment. An FNA of the left sided level VI lymph node in June 2011 tested positive for follicular carcinoma (Figures 3(a)–3(c)).

**Figure 4:**
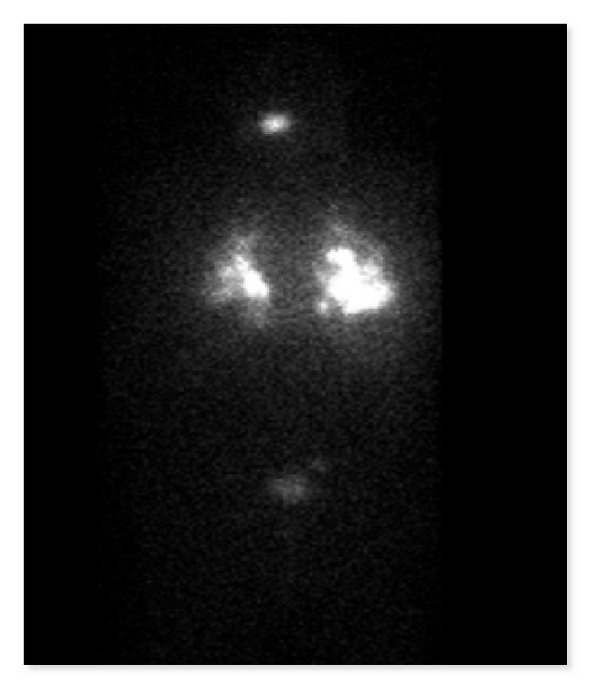
Unstimulated I-131 scan on March 28, 2012.

**Figure 5:**
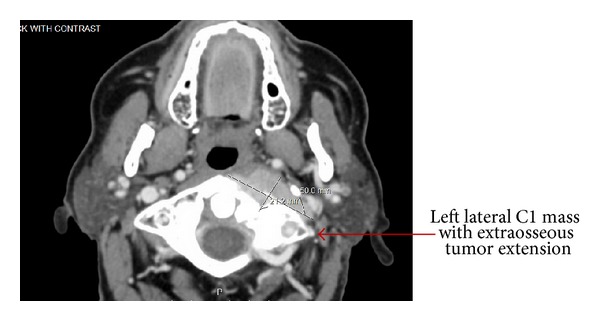
CT scan of left cervical C1 mass.

Levothyroxine was restarted after the TSH level reached the normal range 1.23 mIU/mL by November, 2011. It was discontinued again in preparation for treatment with I-131; however, after three months off of levothyroxine, TSH did not return to levels adequate enough for a repeat radioiodine therapy. Diagnostic whole body scan without rhTSH stimulation was strongly positive in lungs and pelvis fourteen months after initial I-131 treatment (Figure 4). He received another treatment on April 17, 2012, with 210 mCi. A whole body scan 4 months later showed increased radiotracer uptake in the anterior right 6th rib and a CT of the soft tissue and neck showed a large destructive lesion to cervical C1 vertebrae (Figure 5). The thyroid labs after the second dose of I-131 were TSH 10.34 mIU/mL; FT4 0.51 ng/dL; and thyroglobulin 8318 ng/mL.  Table 1 shows the TSH, FT4, and thyroglobulin levels from November 2011 to March 2014.

**Figure 6:**
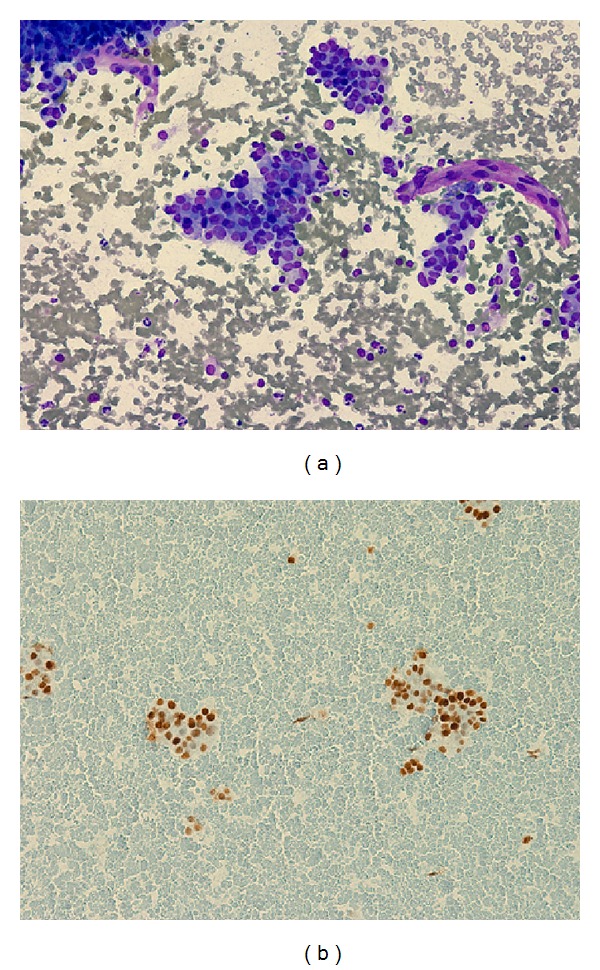
(a) C1 vertebrae biopsy positive for metastatic follicular carcinoma. (b) C1 vertebra biopsy positive for metastatic follicular carcinoma.

**Figure 7:**
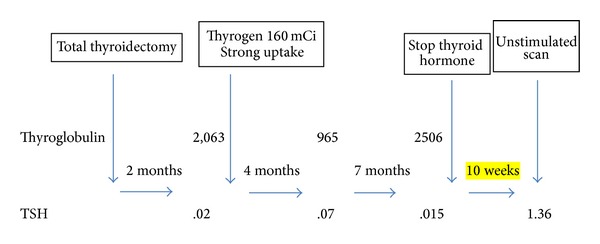
Changes in thyroglobulin and TSH values during course of treatment. Normal values: TSH (thyroid stimulating hormone) 0.34–5.6 *μ*IU/mL; thyroglobulin 1.6–50 ng/mL.

A CT exam of the neck in June 2012 showed an increase in the soft tissue mass and a CT guided cervical (C1) spine FNA was consistent with metastatic follicular thyroid cancer (Figures 6(a) and 6(b)).

The T4 production in this patient was from the metastatic thyroid tissue that had spread to thyroid bed, lungs, sacrum, and pelvis. Treatment with an investigational tyrosine kinase inhibitor was begun with greater than 30% response in metastatic lesions and a drop in thyroglobulin. His lesions remained stable 13 months after initiation of therapy.

## 4. Discussion

The cases regarding differentiated thyroid cancers producing thyroid hormone to cause hyperthyroidism and thyrotoxicosis or maintaining euthyroid state remain quite rare. Ikejiri et al. described a case of hyperthyroidism from functional bone metastasis (posttotal thyroidectomy, I-131 ablation, and transcutaneous intra-arterial embolization therapy) requiring methimazole to prevent thyrotoxicosis [5]. Radioactive iodine (I-131) ablation therapy, in conjunction with antithyroid medication, has been successfully used in treating thyrotoxicosis and also in decreasing the size of the metastatic lesions with remission rates of approximately 33% [6].

Paul and Sisson noted that an increase in FT3 with normal FT4 is a common occurrence, thus emphasizing the importance of measuring a FT3 level in working up a case of thyrotoxicosis [7]. They also noted that the response to I-131 therapy correlated well with outcomes, and the survival rates in metastatic disease with thyrotoxicosis were similar to patients with metastatic disease who were euthyroid [7].

Similar to the patient presented in our case report, Boucher et al. described a metastatic follicular cancer case wherein a patient remained euthyroid for 3 months after discontinuing oral T4 therapy in preparation for a total body scan [8]. The patient had undergone radical thyroidectomy years earlier and an I-131 scan confirmed the absence of thyroid tissue and demonstrated uptake in the skull, sacrum, pelvis, and lungs. The authors reasoned that tumor metastasis was at least partially TSH dependent and T3 administered indeed suppressed TSH production. However, despite several I-131 therapies (total 840 mCi) the tumor growth persisted and led to the patient's death [8]. The authors acknowledge that it remains unclear how the same tumor can be both TSH dependent and autonomous. A possible explanation offered by the authors is that two distinct groups of cells with different degrees of differentiation exist in the same tumor: one which is TSH dependent and the other which is not hormonally controlled, the latter of which contributed to the growth of the tumor.

An alternate explanation is that the same cell type became progressively less differentiated as the tumor progressed, losing its ability to take up iodine, thus ceasing the potential to produce thyroid hormone and gaining the ability to resist I-131 therapy [8]. In either case the tumor cells which were differentiated enough to retain a quasinormal thyroid production may have perished with radioiodine ablation therapy and the undifferentiated cells persisted and multiplied. Well-differentiated tumors have a better prognosis compared to poorly differentiated ones and the latter is seen more often in cases of late metastasis and is associated with absence of I-131 uptake [8].

The pathogenesis of hormone production with resulting hyperthyroidism by metastatic follicular cancer remains largely unknown but a few mechanisms have been proposed. Paul and Sisson suggested that thyroid stimulating immunoglobulin (TSI) may play a role in the production of thyroid hormone by the direct stimulation and resulting growth of metastatic thyroid carcinoma [7]. Ottevanger et al. support the viewpoint that endogenous TSH stimulation may be responsible for enhancing the production of thyroid hormone by metastatic thyroid carcinoma [9]. They described a patient who underwent total thyroidectomy and radioiodine ablation but posttreatment did not require levothyroxine therapy. The metastatic lesions were producing thyroid hormone and the TSH was suppressed during this replacement-free period. The TSH continued to fall while the patient was off therapy with a rise in T4 and T3 levels. It was not until after the thoracic wall lesion, thought to be responsible for the bulk of the hormone production, was removed and a second radioiodine was administered that TSH began to rise in concert with lowering T3 and T4 levels. However, during a later replacement-free period, the TSH began to rise but this time the T3 and T4 levels also increased demonstrating TSH dependency. After the 6th I-131 therapy, T3 and T4 levels finally began to decline. Initially the T3 and T4 levels were similar but during the course of the disease the ratio of T3 to T4 started to increase as the T4 levels began to dwindle; yet T3 levels remained steady. To explain this effect, Ottevanger et al. conducted in vitro studies which showed an impaired iodine uptake and iodination in thyroid cancer [9]. The authors hypothesize that the increase in amount of T3 production is lent to in part to the amount of “dedifferentiation” and subsequent impaired iodine uptake leading to more monoiodotyrosinase production compared to diiodotyrosinase by the tumor.

Lang and Flesch describe a patient with advanced metastatic follicular thyroid cancer with elevated T3 levels causing clinical symptoms of hyperthyroidism but normal to low T4 levels [10]. They proposed that environmental causes, particularly iodine deficiency, may play a role in thyroid hormone production by metastatic differentiated thyroid carcinoma. They conducted a cross-sectional study on 125 patients who were subjected to a total or near total thyroidectomy and were followed periodically for the presence of metastases. 35 patients subsequently were found to have distant metastasis and 6 out of the 35 subjects showed evidence of active thyroid hormone production. The demographic, lab, and clinical features of these 6 patients who showed active hormone production were strikingly similar. All six had the follicular type of thyroid carcinoma, a history of long-standing goiter as the first presentation of their disease, and an increased serum thyroglobulin compared to the nonhormone producing subjects. Not surprisingly, 5 of these 6 subjects had come from an iodine-deficient area, which explains the higher prevalence of goiter. From the results of their cross-sectional study the authors concluded that long-standing goiter and iodine deficiency may have contributed to the slow growth of the metastatic lesions that retained hormone producing ability [10].

In order to understand the mechanism of tumor metastasis by the dedifferentiated thyroid tumor cells, scientists have focused on cancer cell membrane structures. Several studies have analyzed the role of cell membrane proteins in association with tumor aggressiveness and distant metastasis. These include E-cadherin, a glycoprotein involved in cell epithelial integrity, and Beta-catenin, a protein involved in cell adhesion.

A decrease or absence of E-cadherin expression has been implicated in separate studies to increased tumor aggressiveness and decreased survival [11]. Brecelj et al. examined the prognostic value of E-cadherin expression in follicular carcinoma of the thyroid and its variants (Hurthle cell and Insular) [11]. The authors found that reduced expression of E-cadherin, as defined by membranous labeling of 90% of tumor cells, was considerably more common in widely invasive tumors (10/20), as compared to minimally invasive ones (5/34). These findings were replicated by Kato et al. with more convincing results [12]. They reported that all 4 of the widely metastatic tumors displayed reduced E-cadherin staining while only 2 of the 16 minimally invasive follicular carcinomas met the reduced E-cadherin criteria. Brecelj et al. assert that E-cadherin, in addition to a predictor of tumor invasiveness, is an independent predictor of subsequent metastatic spread.

Another important protein that is involved in tumor differentiation and metastasis is Beta-catenin. This protein normally found on the cell membrane plays an important role in cell adhesion and signal transduction [13]. Garcia-Rostan et al. analyzed the role of this protein in follicular thyroid tumor progression and metastasis [13]. The authors concluded that a decrease in B-catenin expression in thyroid tumor cell membrane correlated with a progressive loss of tumor differentiation and this reduction was found to be significantly greater in follicular carcinomas when compared to follicular adenomas. The authors report that this reduction of the cell adhesion protein is directly linked with the tumor's ability to separate from its primary site and metastasize to the blood vessels and various other distant sites [13].

## 5. Conclusion

This case is exceptional because the aggressive nature of this tumor, demonstrated by the metastases and resistance to I-131 therapy, goes against the typical behavior of dedifferentiated tumors. The metastatic lesions continued to secrete thyroid hormone and remained radioiodine avid despite rapid progression after I-131 therapy. Future studies focused on the physiology of thyroid hormone production by metastatic well-differentiated thyroid cancers need to be conducted in order to determine the exact mechanism of this rare phenomenon.

## Figures and Tables

**Table 1:** Thyroid function tests and thyroglobulin levels from Nov. 2010 to Mar. 2014.

Visit date	TSH (*µ*IU/mL)	FT4 (ng/dL)	Thyroglobulin (ng/mL)
Nov. 3, 2010	0.023	2.13	2063
Jan. 12, 2011	0.077	2099	965
Jul. 13, 2011	0.015	1.94	2506
Nov. 2, 2011	1.23	0.72	5019
Mar. 7, 2012	0.37	0.89	5178
Jun. 6, 2012	10.34	0.51	8318
Mar. 12, 2014	0.35	1.55	2632

Abbreviations and normal values: FT4 (free T4) 0.58–1.64 ng/dL; FT3 (free T3) 2.5–3.9 pg/mL; TSH (thyroid stimulating hormone) 0.34–5.6 *µ*IU/mL; thyroglobulin 1.6–50 ng/mL.
